# Correction: Chiral hydroxymethyl-1*H*,3*H*-pyrrolo[1,2-*c*]thiazoles: the search for selective p53-activating agents for colorectal cancer therapy

**DOI:** 10.1039/d5md90004b

**Published:** 2025-02-03

**Authors:** Mees M. Hendrikx, Adelino M. Pereira, Ana B. Pereira, Carla S. C. Carvalho, João L. P. Ribeiro, Maria I. L. Soares, Lucília Saraiva, Teresa M. V. D. Pinho e Melo

**Affiliations:** a University of Coimbra, Coimbra Chemistry Centre – Institute of Molecular Sciences and Department of Chemistry 3004-535 Coimbra Portugal tmelo@ci.uc.pt; b LAQV/REQUIMTE, Laboratório de Microbiologia, Departamento de Ciências Biológicas, Faculdade de Farmácia, Universidade do Porto Porto Portugal

## Abstract

Correction for ‘Chiral hydroxymethyl-1*H*,3*H*-pyrrolo[1,2-*c*]thiazoles: the search for selective p53-activating agents for colorectal cancer therapy’ by Mees M. Hendrikx *et al.*, *RSC Med. Chem.*, 2024, **15**, 1652–1663, https://doi.org/10.1039/D4MD00076E.

Adelino M. R. Pereira was listed as one of the authors in the above-mentioned published article. This is to inform the readers that their name has now changed to Adelino M. Pereira.

The Royal Society of Chemistry apologises for these errors and any consequent inconvenience to authors and readers.

